# Prevalence of Iodine Deficiency among School Children from New Settlement in Kyrgyzstan

**DOI:** 10.3390/children8090817

**Published:** 2021-09-16

**Authors:** Begaiym Urmatova, Hyunsook Shin, Soonyoung Shon, Zeinep Abdyldayeva, Elmira Ishaeva, Valeriya Knyazeva

**Affiliations:** 1 Department of Faculty Pediatrics, Kyrgyz State Medical Academy, 92 Akhunbayev St, Bishkek 720020, Kyrgyzstan; begaimkubanychbek@gmail.com (B.U.); zaken0746@mail.ru (Z.A.); ishaeva63@mail.ru (E.I.); peach.lis.87@mail.ru (V.K.); 2 College of Nursing Science, Kyung Hee University, Seoul 02447, Korea; 3 College of Nursing, Keimyung University, Daegu 42601, Korea; sy.shon@kmu.ac.kr

**Keywords:** child health, goitre, iodine deficiency, school children, screening

## Abstract

This study assesses the status of iodine deficiency among at risk-children and adolescents living in migrant settlements in the Kyrgyz Republic. Children aged 7–15 years from two regional primary schools in the new settlement regions were screened for cognitive and behavioural signs of iodine deficiency using questionnaires. The functional state of the thyroid gland was assessed using ultrasonography and blood tests. Out of 1058 schoolchildren, 15.8% showed signs of iodine deficiency. Female children aged 10–12 years showed a higher prevalence of iodine deficiency. The families of schoolchildren reported limited use of seafood and iodised salt. Children in the migrant regions were at risk of iodine deficiency disorder. Among children, clinical manifestations of iodine deficiency were observed as negative hormonal levels or the presence of goitre. Further investigation on standardised screening instruments for iodine deficiency and the relationship among multilevel analyses are warranted.

## 1. Introduction

During the last decade, micronutrient deficiencies, including that of iodine, iron, and vitamins, have been a growing health issue among children and adolescents worldwide. Iodine deficiency remains a health concern, even though international collaborative approaches such as food fortification and supplementation have been successfully implemented globally. According to the World Health Organization (WHO), approximately 1.88 billion of the world’s population are at risk of iodine deficiency. It is the most common and, coincidentally, the only preventable cause of intellectual disability [1]. Approximately 50 million people worldwide experience clinical manifestations arising from low iodine content in food [2]. It is well known that children born from mothers with severe iodine deficiency have serious neurological disorders, such as mental retardation [3]. 

Iodine deficiency in the body leads to the development of goitre. Endemic goitre is a disease characterised by progressive enlargement of the thyroid gland with different functional activities. The disease is observed in areas where the soil, and therefore, water and food are poorly iodised. The iodine content in seawater is ~50 mcg/L, which volatilises into the atmosphere and is returned to the soil by rain slowly and is often incomplete [4]. 

At the end of the 20th century, with the efforts of the International Council for the Control of Iodine Deficiency Diseases (ICCIDD) of the WHO and several other organisations, the problem of iodine deficiency was recognised as a global and social concern for humanity. In 1994, the ICCIDD recommendations on the use of iodine deficiency indicators came into force, and criteria for its severity were developed. The most recognised method of mass iodine deficiency prevention is the iodisation of dietary salt, which can reach the entire population of the iodine-deficient region [5].

Similar to most mountainous regions of the world, iodine deficiency in soil, water, and food is endemic in the Kyrgyz Republic. The lack of iodine in nature causes the development of several iodine deficiency disorders in the population, which leads to the development of diseases such as endemic goitre and hypothyroidism. The ‘hidden’ consequences of iodine deficiency include female infertility, reproductive disorders, miscarriages and stillbirths, increased mortality of children in the first year of life, physical and mental retardation in children, deaf-mutism, squint, dwarfism, and congenital anomalies [2,6]. Therefore, the National Program for the Prevention of Iodine Deficiency Disorders was implemented to reduce the prevalence of disorders in the Kyrgyz Republic between 2010–2014. However, the program mostly focused on at-risk groups, such as pregnant and lactating women [7].

The increasing prevalence of iodine deficiency in the country determines the relevance of the problem and necessitates the study of iodine deficiency among schoolchildren [8]. Currently, international development agencies support the introduction of iodised salt and identification of iodine deficiency. However, international support has been limited in addressing the iodine status of schoolchildren in recent years. The new settlement regions in the capital city of the Kyrgyz Republic were established during the independence period from the Soviet Union. People from rural areas or Russia lived in the border areas of the capital city, and hence, a higher rate of residents were not registered in the current residence. Children in these regions demonstrated a high prevalence of preventable diseases such as anaemia and iodine deficiency disorder due to limited resources and healthcare support [9]. 

The purpose of this study was to investigate the prevalence of iodine deficiency among schoolchildren and adolescents living in the migrant settlements. A multi-assessment approach focusing on iodine deficiency, growth state, risk factors, cognitive function tests, and functional state tests was used. 

## 2. Materials and Methods

### 2.1. Study Design

A descriptive cross-sectional study design was used to identify and assess iodine deficiency among schoolchildren. Data were collected between December 2018 and March 2019.

### 2.2. Participants

Participants (*n* = 1058; girls = 558, boys = 500) were school children aged 7–15 years and their parents living in the new settlements and attending primary schools located in a residential area of Ak-Orgo in Bishkek. All children included in the study were divided into three groups depending on their age. The first age group consisted of 407 children aged 7–9 years; the second group, 325 children aged 10–12 years; and the third group, 326 children aged 13–15 years.

### 2.3. Ethical Considerations

The study was approved by the Kyrgyz National Institutional Review Board (No. 7). A written informed consent form was signed by the caregivers of the participating children before study enrolment. Children and caregivers could withdraw from the study at any time without further obligation, and their participation was voluntary.

### 2.4. Measurement

**Figure 1:**
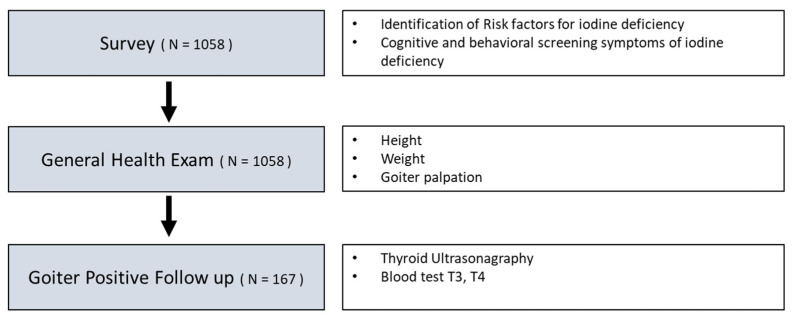
Research flow.

Figure 1 shows the research flow. We surveyed parents and children to identify risk factors for iodine deficiency and screened for cognitive and behavioural signs of iodine deficiency. The questionnaire consists of 13 items including risk factors and signs of iodine deficiency. Signs of iodine deficiency were assessed based on the presence of fatigue, declined memory and school performance, and decreased concentration and attention. Students were instructed to complete the survey questions with their parents. 

All participants underwent general health examinations, including head-to-toe physical examination, as well as assessment of growth and development. Palpation of the thyroid gland for measurement of thyroid size was performed by paediatricians at the school. Palpable goitre was staged based on WHO criteria: grade 0 (no palpable or visible goitre), grade 1 (palpable, but not visible when the neck is in the normal position), grade 2 (visible goitre when the neck is in the normal position) [6]. Additional examinations for children with enlarged thyroid (goitre) were performed in university affiliated hospitals where those diagnostic procedures including ultrasonography and blood tests were available. Children with goitre underwent ultrasonography, using the Aloka SSD900 apparatus, as well as blood tests to assess the functional state of the thyroid gland. Blood tests were performed to assess the serum levels of free T4 and the thyroid-stimulating hormone (TSH). Weight was measured using Tanita TBF5383611, and height was measured using a cardboard attached to the wall.

### 2.5. Statistical Methods

Data were analysed using SPSS version 23.0(IBM Inc., New York, NY, USA). Findings from general health examinations, cognitive tests, and functional tests were analysed using descriptive statistics. Growth states with height and weight were identified using growth curve and Z scoring. The state scores demonstrating iodine deficiency were analysed according to the general characteristics of the participants, and the results were analysed using chi-squared test.

## 3. Results

### General Characteristics of Participants

In the survey on risk factors and signs of iodine deficiency, parents of school children reported they rarely consumed seafood (35.9%) and iodised salt (17.8%). Symptoms of fatigue, weakness, and drowsiness were reported in 14.7% of the children, whereas 28.3% reported weak concentration and memory and a decrease in school performance. Frequent colds were observed in 12.2% of the children, visible bulge at the base of the neck in 8.7%, dry skin and hair in 14.4%, brittle nails and excessive weight gain in 9.6%, and weight loss in 7.9%.

Table 1 shows the general characteristics of participants. Among children in the age group of 7–9 years, 18.8% were boys and 19.7% were girls; among 10–12 years, 13.4% were boys and 17.3% were girls; and among 13–15 years, 15.0% were boys and 15.8% were girls. 

Table 2 shows the participants’ growth and self-perceived symptoms of iodine deficiency based on age groups. A total of 2.9% (*n* = 31) of children in the study had stunting, indicated by z scores less than two. In addition, 7.2% (*n* = 76) of the children were underweight, also indicated by z scores less than two. 

Table 3 shows the number of participants with enlarged thyroids and further examination results among children with goitre. 

A total of 15.8% of the participants (*n* = 167) presented with an enlarged thyroid. No child had grade 2 goitre. When we analysed the prevalence of goitre according to the age group, children aged 10–12 years showed the highest percentage of goitre prevalence (44.3%, *n* = 74), followed by children aged 7–9 years (35.3%, *n* = 59) and those aged 13–15 years (20.4%, *n* = 34). 9.6% of the participants with goitre showed stunting while 4.2% of them were underweight.

Children with goitre underwent an ultrasound examination of the thyroid gland. They showed normal volume of thyroid gland, smooth transition angle of the isthmus into the lobes, and normal range of echogenicity. A few children (*n* = 3) showed slightly reduced echogenicity but not out of the age norm. However, most children showed unchanged echogenicity. To measure hormone levels among children with goitre, 94 children in the study with enlarged thyroid underwent blood tests, which revealed normal levels of thyroid hormones and TSH according to the age norm. 

Cognitive functions of children with enlarged goitre assessed by the survey questions on the presence of signs revealed increased fatigue, decreased concentration and attention, and decreased memory and school performance across all age groups. In the younger age group (7–9 years), 50 children (out of 59) with goitre, experienced rapid fatigue and decreased concentration, memory, and school performance. Among children aged 10–12 years, 62 children (out of 74) experienced increased fatigue, instability, had decreased ability to concentrate, and decreased performance. In the 13–15 years age group, 29 (out of 34) children showed significantly reduced attention.

## 4. Discussion

A significant number of children in the present study had enlarged thyroid and goitre. According to previous studies on children in the same region [10], the prevalence rate of goitre among children in this age group is 20.1%. Children aged 10–12 years had the highest prevalence of goitre, resulting that this age group was the most at risk for goitre. Further investigation of differences among the study participants in this age group is necessary. In addition, a higher prevalence rate was observed among the girls, which is consistent with the findings of a study in South Tajikistan [10]. In a recent Romanian study [11], children residing in mountainous areas demonstrated no gender differences in goitre presence. However, regardless of place of residence, goitre was more common among girls at puberty than in boys except a reverse finding in an Indian study presenting more prevalence of goitre in boys than girls [12].

Children in this study reported increased fatigue, decreased concentration and attention, and decreased memory and school performance across all age groups; when we examined children with enlarged thyroids, they showed age-normal ranges of ultrasonography results and hormonal levels. Positive goitre and associated cognitive findings with a normal range of functional thyroid states suggests that there were certain gaps between screening test results and diagnostic study results. According to the WHO clinical guidelines on iodine deficiency and iodine deficiency disorder [13], indicators such as prevalence of goitre measured by palpation, ultrasound, and median urinary iodine concentration are recommended to identify risk factors for school-aged children.

A total of 2.9% of the children in the study had stunting, and 7.2% were underweight, indicated by z scores less than two. Children with goitre showed a higher prevalence of stunting and being underweight as suggested from previous studies [14,15]. Further analysis revealed that stunting showed a significant relationship (r = 0.342, *p* < 0.001) with being underweight. Considering that the physical development of 38.5% of children living in high altitudes in eastern Europe showed abnormal findings with low intellectual development, children in the present study showed relatively low rates of physical development disturbance [11]. However, findings showed a relationship between goitre presentation and low physical development.

The Kyrgyz National Salt Iodization Program was launched in 2001. Notably, iodised salt became available in the new settlement area, but a third of participants in this study reported that their households did not use iodised salt. Furthermore, 15.8% of the participants had goitre, which was relatively high compared to nearby countries such as Ethiopia (4.2%), following their national salt iodization program [16]. Testing small sample of salt from house to school to identity any iodine content in salt could be helpful to identify the study area’s risk of iodine deficiency. Even though children with goitre demonstrated normal levels in their ultrasonography findings and related blood tests, more studies on adequate intake in at-risk populations and use of urine tests for iodine deficiency are required [17]. However, recent studies [18] have addressed the consequences of excessive iodine intake and possible adverse effects in places where iodine deficiency has previously occurred.

This study was performed using a multi-assessment approach focusing on iodine deficiency, growth state, risk factors, cognitive function tests, and functional state tests. Previous studies have been conducted focused on either screening or diagnostic tests, which resulted in decreased (weak) specificity and sensitivity (accuracy of the specific test). Considering that Sunamak and Duren [19] reported that thyroid ultrasonography is a reliable method to measure thyroid and that careful physical examination is needed to detect nodules in the thyroid, this stepwise approach to iodine deficiency evaluation is valuable as it informs conducting of more reliable tests, specifically in communities requiring careful assessment. Further studies using the thyroid ultrasonography for screening young children whose thyroid glands are small are warranted to explore more accurate findings.

### Limitations

This study has a few limitations. The present study was performed in a single region of Kyrgyz, resulting in limited representation. In addition, the study did not use indicators such as urinary iodine and iodine concentrations of the consumed foods. Further studies with a larger sample size indicating clinical manifestations may be warranted to gain a deeper understanding of iodine deficiency in the region.

## 5. Conclusions

Based on the higher prevalence of goitre in the study area, Kyrgyz children could still be at risk of iodine deficiency. The multilevel analysis of these children and adolescents indicates that clinical manifestations of iodine deficiency can be observed in children, demonstrating the presence of goitre. The limited relationship between the presence of goitre and diagnostic studies of hormonal level changes or ultrasonography has indicated important measurement issues. Further investigation on standardised screening instruments for iodine deficiency and the relationship among multilevel analyses are necessary.

## Figures and Tables

**Table 1:** General characteristics of participants (*n* = 1058).

Items	Category	*n*	%	Mean(SD)	Min–Max
Sex	Boy	500	47.3		
	Girl	558	52.7		
Age	7–9	407	38.5	10.7 (2.64)	7–15
	10–12	325	30.7		
	13–15	326	30.8		
Weight (kg)	Z score ≤ −2	76	7.2	32.8 (6.37)	21–60
Height (cm)	Z score ≤ −2	31	2.9	142.66 (12.24)	120.6–167.2

**Table 2:** Participants’ growth based on age groups (*n* = 1058).

Age Group	Gender	*n*	BMI Percentile	SD	BMI z-Score	SD	Height (cm)	SD	Stunted: Z Score < −2 (*n*)	*X* ^2^	Weight (kg)	SD	Underweight: Z Score < −2 (*n*)	*X* ^2^	Signs of Iodine Deficiency (Mean, SD)	Signs of Iodine Deficiency (Median, SE)
7–9 years	M	199	29.7	30.12	0.32	0.60	129.9	4.72	0		27.6	2.09	0		1.9 (1.52)	2 (0.10)
	F	208	23.8	28.51	0.21	0.52	129.5	4.94	0		27.2	1.88	0		2.2 (1.70)	2 (0.11)
	subtotal	407	26.7	29.42	0.26	0.56	129.7	4.83	0		27.4	2.00	0		2.0 (1.62)	2 (0.08)
10–12 years	M	142	1.9	4.03	−1.04	0.79	146.1	5.48	2		33.3	2.36	0		2.6 (1.66)	3 (0.13)
	F	183	2.7	5.59	−1.02	0.80	145.9	5.43	5		33.1	2.27	2		2.6 (1.87)	3 (0.13)
	subtotal	325	2.3	4.98	−1.03	0.79	146.0	5.45	7		33.2	2.31	2		2.6 (1.78)	3 (0.09)
13–15 years	M	159	0.6	2.10	−1.61	1.35	154.2	6.93	20		38.7	6.42	44		3.0 (1.82)	3 (0.14)
	F	167	0.2	0.62	−1.64	1.35	156.8	5.87	4		39.8	6.72	30		3.1 (1.55)	3 (0.12)
	subtotal	326	0.4	1.54	−1.62	1.35	155.5	6.53	24		39.2	6.59	74		3.1 (1.6)	3 (0.09)
Total		1058	11.1	22.20	−0.72	1.24	142.7	12.24	31 (2.9%)	<0.001	32.8	6.37	76 (7.2%)	<0.001		2 (0.05)

M = male; F = female.

**Table 3:** Clinical characteristics of school children with enlarged thyroids (*n* = 167).

Age Groups		*n*	%	Height (cm)	Stunted: Z Score < −2 (*n*)	*X* ^2^	Weight (kg)	Underweight: Z Score < −2 (*n*)	*X* ^2^	TSH (mU/L)	FreeT4 (ng/dL)	Perceived Cognitive Signs of Iodine Deficiency		
												Fast Fatigue	Declined Memory and School Performance	Decreased Concentration and Attention
7–9 years	Boy	16	9.6	130.6 (±5.72)	0		27.4 (±2.21)	0		3.2 (±0.55)	1.2 (±0.16)	13	13	13
	Girl	43	59	131.9 (±4.42)	0		27.8 (±1.43)	0		2.8 (±0.68)	1.7 (±1.53)	37	37	37
	Subtotal	59		131.5 (±4.79)	0		27.7 (±1.66)	0		2.9 (±0.65)	1.6 (±1.29)	50	50	50
10–12 years	Boy	36	21.6	145.9 (±5.21)	1		33.2 (±1.77)	0		2.9 (±0.54)	1.4 (±0.26)	32	32	32
	Girl	38	22.8	144.2 (±4.98)	2		31.9 (±1.84)	1		3.1 (±0.49)	1.3 (±0.23)	30	30	30
	Subtotal	74		145.0 (±5.12)	3		32.5 (±1.92)	1		3.0 (±0.52)	1.4 (±0.25)	62	62	62
13–15 years	Boy	15	9.0	152.1 (±4.25)	0		35.4 (±3.38)	8		3.0 (±0.30)	1.4 (±0.27)	11	11	11
	Girl	19	11.4	146.2 (±9.84)	4		35.1 (±2.51)	7		3.0 (±0.41)	1.5 (±0.20)	18	18	18
	Subtotal	34		148.8 (±8.31)	4		35.3 (±2.88)	15		3.0 (±0.34)	1.5 (±0.24)	29	29	29
Overall		167		141.0 (±9.21)	7(4.2%)	0.024	31.4 (±3.55)	16(9.6%)	<0.001	2.97 (±.54)	1.47 (±0.82)	141	141	141

## Data Availability

Data could be provided when requested.
